# Employment of Alcohol in Health and in Disease

**Published:** 1878-08

**Authors:** 


### The Employment of Alcohol in Health and in Disease.

Renewed and very serious attention has been of late paid, by the medical men of Great Britain and Ireland, to the subject of the use and abuse of intoxicants. Though the vice of drunkenness is bad enough in this country, it is worse in those islands. The annual cost of the alcoholic liquors consumed amounts to the enormous sum of *seven hundred and thirty million dollars;* the number of inmates of workhouses who have been brought there by this indulgence is estimated at five-sevenths of the whole; and of the disease, suffering, mortality and deaths indirectly traceable to the same cause only approximate yet most startling, estimates have been made.

The President of the Ulster County Medical Society, Ireland, in contemplating this enormous expenditure, exclaims:—

"This sum is enough, in little over five years, to pay off the national debt; enough, if spent in supplying food, clothing, domestic requirements, and otherwise beneficially, to develop good trade, the physical, mental and moral well-being of the people, national greatness and general prosperity—an average of £23 a year for each family! of nearly £5 for each man, woman and child! And what do the people get in exchange for all this expenditure? Misery, misfortune, disease, crime!"

After a very full discussion, the Society of which he is President declared their opinion that alcholic stimulants are not only unnecessary in health, but are generally hurtful, and therefore, should be discountenanced by medical men.

That apostle of total abstinence, Dr. B. W. Richardson, of London, has been urging the same general facts, by a series of scientific demonstrations and lectures. He does not, however, condemn its use in disease, though he assigns it no prominent place in the materia medica. It does reduce the animal temperature in fever, but the after effects are often most unpleasant and dangerous. The skillful exhibition of alcohol in certain cases of nervous disease is productive of benefit. It is alleged to be a useful antiseptic, and it has been said to be a conservative remedy, partially doing away with the need of food.

He maintains, however, that we must accept the evidence of the value of alcohol in regard to its sustaining power *sub judice*, and when we come to practical concerns, where there can be no doubt whatever, we must be even more reserved. In the treatment of starvation in cases of stricture of the œsophagus, the very types of cases of the kink under consideration, no more injurious practice can be applied than the administration of alcohol by any means. In such instances alcohol excites the heart, produces febrile disturbance, interferes with the natural secretions, increases cough, and encourages waste. By the side of milk it has no place whatever, neither does it offer a single indication of its value as a food.

In studying the relation of alcohol to disease, no other arguments can, in the opinion of Dr. Richardson, be scientifically offered in support of its use and service, beyond those to which he has drawn attention. Excepting in the cases named, in which alcohol may be supposed to play a useful part, but in which it cannot be placed as standing alone and as an absolute necessity, he would maintain that total abstinence is as sound a practice in disease as it is in health. Certainly no facts, no series of facts, are more remarkable than those which are revealed when the treatment of disease is observed without the use of alcohol as a remedy. It soon comes to be a rule with all observers in this school of thought, that the less they trust to alcohol the less they see reason to believe in it; that even in its position as a medicine it is of all the more useful remedies the least necessary; and that he who willfully leaves its use to the indiscriminate choice of the sick, or to their ignorant attendants, commits a breach of trust which no professional protection, in this day of awakening knowledge, can for any long time excuse, justify, or guard.—*Med. and Surg. Reporter.*

